# Supplementary material for: A novel mechanism of bulk cytoplasmic transport by cortical dynein in Drosophila ovary
Source: eLife. 2022 Feb 16;11:e75538. doi: 10.7554/eLife.75538 (PMC8896832; doi:10.7554/eLife.75538)
Supplement: Figure 5—source data 1. [file elife-75538-fig5-data1.zip › Figure 5-source data 1/Figure 5-source data 1 legend.docx]

Figure 5-source data 1 contains the original raw pulldown blot (1) and labeled pulldown blot (2), corresponding to the pulldown blot shown in Figure 5B.
