# Supplementary figures and images for: A novel mechanism of bulk cytoplasmic transport by cortical dynein in Drosophila ovary
Source: eLife. 2022 Feb 16;11:e75538. doi: 10.7554/eLife.75538 (PMC8896832; doi:10.7554/eLife.75538)

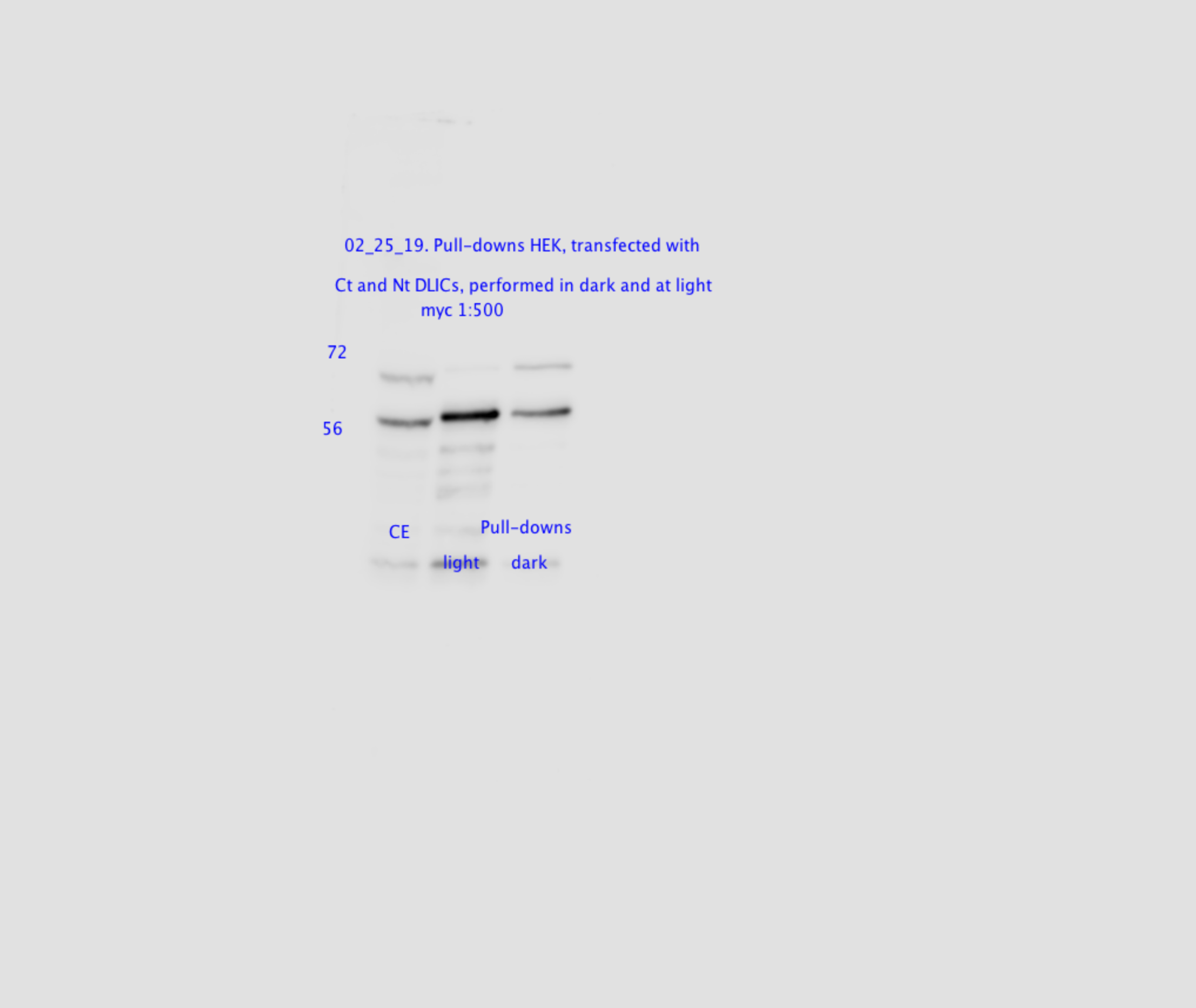

Supplement: Figure 5—source data 1. [file elife-75538-fig5-data1.zip › Figure 5-source data 1/Figure 5-source data 1 (2) labeled pulldown blot.tif]
